# Genes2FANs: connecting genes through functional association networks

**DOI:** 10.1186/1471-2105-13-156

**Published:** 2012-07-02

**Authors:** Ruth Dannenfelser, Neil R Clark, Avi Ma'ayan

**Affiliations:** 1 Department of Pharmacology and Systems Therapeutics, Systems Biology Center of New York (SBCNY), Mount Sinai School of Medicine, One Gustave L. Levy Place, Box 1215, New York, NY, 10029, USA

## Abstract

****Background**:**

Protein-protein, cell signaling, metabolic, and transcriptional interaction networks are useful for identifying connections between lists of experimentally identified genes/proteins. However, besides physical or co-expression interactions there are many ways in which pairs of genes, or their protein products, can be associated. By systematically incorporating knowledge on shared properties of genes from diverse sources to build functional association networks (FANs), researchers may be able to identify additional functional interactions between groups of genes that are not readily apparent.

****Results**:**

Genes2FANs is a web based tool and a database that utilizes 14 carefully constructed FANs and a large-scale protein-protein interaction (PPI) network to build subnetworks that connect lists of human and mouse genes. The FANs are created from mammalian gene set libraries where mouse genes are converted to their human orthologs. The tool takes as input a list of human or mouse Entrez gene symbols to produce a subnetwork and a ranked list of intermediate genes that are used to connect the query input list. In addition, users can enter any PubMed search term and then the system automatically converts the returned results to gene lists using GeneRIF. This gene list is then used as input to generate a subnetwork from the user’s PubMed query. As a case study, we applied Genes2FANs to connect disease genes from 90 well-studied disorders. We find an inverse correlation between the counts of links connecting disease genes through PPI and links connecting diseases genes through FANs, separating diseases into two categories.

****Conclusions**:**

Genes2FANs is a useful tool for interpreting the relationships between gene/protein lists in the context of their various functions and networks. Combining functional association interactions with physical PPIs can be useful for revealing new biology and help form hypotheses for further experimentation. Our finding that disease genes in many cancers are mostly connected through PPIs whereas other complex diseases, such as autism and type-2 diabetes, are mostly connected through FANs without PPIs, can guide better strategies for disease gene discovery. Genes2FANs is available at: http://actin.pharm.mssm.edu/genes2FANs.

## Background

Studies that utilize genome-wide profiling methods which attempt to explain the differences between two or more experimental conditions such as cells treated with a drug vs. control, diseased tissue vs. normal, gene or protein expression at different time points during cellular differentiation or reprogramming, or candidate gene lists harboring mutations associated with a particular disease, produce lists of genes/proteins without apparent functional relationship. These lists are commonly analyzed using software tools and databases that map genes to known pathways or construct subnetworks that connect input lists of genes using known protein-protein or other types of molecular interactions [1-10]. Such methods have been instrumental for organizing and reusing prior knowledge to understand new high-content experimental results. Prior knowledge networks, in particularly protein-protein interaction networks, have been useful for predicting unknown functions for genes [11,12], new interactions between proteins [13], novel disease genes [14], and guiding experimental research efforts by prioritizing the most likely regulators to test at the bench [15]. The resultant subnetwork diagrams from these analyses are useful because this prior knowledge, displayed as a network diagram, contains information about the relationships between the genes identified experimentally. This approach also abstracts the genes from the query list to higher order biological functions, allowing for the identification of novel relevant genes.

Software tools that provide users the ability to build subnetworks from lists of genes using prior knowledge networks are continually gaining popularity. For instance, a system that we developed a few years ago, Genes2Networks, utilizes twelve protein-protein interaction databases to connect lists of mammalian gene products using a shortest path algorithm [1]. Similarly, the software VisAnt version 3.5 goes a step further to automatically compute enrichment for gene ontology (GO) terms in identified PPI subnetworks [2]. Integrating PPIs, gene regulatory interactions, metabolic networks, and cell signaling networks, ConsensusPathDB provides methods to find connections between human, mouse and yeast genes [3]. Cytoscape, one of the leading academic platforms for building and visualizing networks, through its modular plug-ins, provides ways to construct networks, find paths between nodes, and compute network properties in an integrative manner [16]. Similar functionality is available in PatikaWeb [4], a web application with an underlying large protein interaction database. STRING, arguably the most comprehensive molecular interaction database, contains many different interactions including protein-protein and co-expression with assigned confidence scores [5]. Similar functionality is also available in BioPixie, initially developed for yeast but more recently extended to cover the mouse [17]. Visualization tools such as N-Browse [6], AVIS [18], FNV [19], and Cytoscape Web [20] display subnetworks from heterogeneous types of data sources with different color edges and nodes to represent different types of links and nodes on the web. GeneMANIA [7], another subnetwork generation tool, utilizes Cytoscape Web to display known and predicted protein-protein interactions, co-expression interactions, interactions based on shared pathways, and genetic interactions. So far, most subnetwork building software tools only utilize a few types of prior knowledge networks, mostly protein-protein interactions, co-expression, metabolic, and cell signaling pathway networks. Here we extend on these efforts by generating 14 functional association networks (FANs) from gene set libraries and combine them with a large-scale network of mammalian protein-protein interactions. The FANs were systematically generated by converting gene set libraries to networks, connecting pairs of genes based on their shared functional annotations. These functional association networks (FANs) together with protein-protein interaction networks are our background knowledge database for building and visualizing subnetworks from input lists of genes. Keeping functional relationships separate, we allow users to control what layers of functional associations they wish to integrate into their analysis. This system is delivered as a web based interactive tool called Genes2FANs. To demonstrate the utility of the Genes2FANs approach we applied the software to connect lists of disease genes for 90 diseases that have many known mutated genes. We find an inverse correlation between the number of protein-protein interaction links and the number of functional annotation links identified when connecting lists of disease genes. This inverse correlation separates complex diseases into two classes: those that are protein interaction centric, including many cancers, and those that are functional centric, including complex spectral disorders such as autism and type-2-diabetes.

## Implementation

### **Methods for constructing the functional association networks**

**Figure 1:**
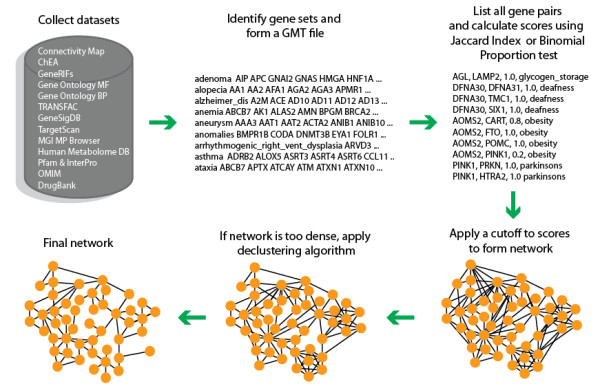
**Process of creating FANs.** The process of creating FANs involves gathering datasets and processing them into GMT files. Using these GMT files, networks are created using either the Jaccard index or a Binomial Proportion test. Large and dense networks are filtered using a declustering method and a cutoff is applied to produce the final FANs.

The first step in assembling the FANs was to gather data spread across a wide variety of databases and online sources. Besides collecting a comprehensive list of available protein-protein and cell signaling networks (see below), we also collected and generated gene set libraries that we later converted to FANs. Gene set libraries store sets of genes in a gene matrix transposed (GMT) file with rows containing a set of genes symbols associated with a given functional term. Using this format we were able to quantify the relationships between pairs of genes based on their co-occurrence membership in sets of the same gene set library using two different similarity measures: the Jaccard index and a Binomial Proportion test. The process of creating FANs from GMT files is outlined (Figure 1).

The Jaccard index is a measure of the similarity of two sets, *A* and *B*, which is given by the ratio of their intersection to their union:

(1) $$J=\frac{A\cap B}{A\cup B}$$

Scores range in values from 0 to 1, where indices of 1 indicate exact similarity and indices of 0 indicate no relation between the sets. In our case, to score similarity between gene pairs, we divided the number of sets for both genes by the number of unique sets each gene belongs to. If we identify the sets *A* and *B* with the set of all lines of the GMT file, in which each of two respective genes are present, the the Jaccard index can be taken as a measure of the degree of association between the genes. The Jaccard index scoring method was applied to gene set libraries (GMT files) that contain a small number of genes per functional term with many different functional terms. Eight FANs were created using this method: miRNAs, mouse phenotypes, metabolites, structural domains, GO biological processes, disease genes, and drug targets. For each network we chose a cutoff that maximizes the tradeoff between coverage (maximizing the number of nodes) and sparseness (minimizing the number of links) (Tables 1 and 2).

**Table 1:** FAN properties

**Network**	**Scoring Method**	**Network Cutoff**	**Data Source**	**Nodes**	**Edges**
CMAP co-expression	Binomial Proportion*	130	Connectivity Map Database	8,924	62,382
Transcription Factors (ChIP-X)	Binomial Proportion*	27	ChEA database	13,223	70,347
GeneRIF	Binomial Proportion*	2000	NCBI GeneRIF	3,777	27,487
GO Molecular Function	Binomial Proportion*	160	Gene Ontology	2,944	23,356
TRANSFAC	Binomial Proportion	27	TRANSFAC	15,252	94,642
GeneSigDB	Binomial Proportion	350	GeneSigDB	10,536	65,776
MicroRNA	Jaccard*	0.3	TargetScan	6,590	46,161
Mouse Phenotype	Jaccard*	0.5	MGI MP Browser	7,553	52,637
Metabolites	Jaccard*	0.35	Human Metabolome Database	3,577	28,617
Structural Domains	Jaccard*	0.5	Pfam and InterPro	6,746	46,463
GO Biological Process	Jaccard*	0.99	Gene Ontology	4,287	29,988
OMIM Expanded	Jaccard	0.99	OMIM Morbid Map	2,051	23,191
OMIM Disease	Jaccard	0.99	OMIM Morbid Map	1,618	22,643
Drug Target	Jaccard	0.5	DrugBank	2,121	16,807
PPI	None	N/A	13 Databases	15,548	64,741

Properties of all the FANs along with their scoring method, scoring cutoff, data source, edge and node totals. * indicates that the declustering method was applied.

**Table 2:** Declustering Details

**Network**	**Declustering Constant (Iterations)**	**Nodes Before**	**Nodes After**	**Edges Before**	**Edges After**
CMAP co-expression	2,000	8,924	8,924	119,420	61,362
Transcription Factors (ChIP-X)	1,500	13,223	13,223	110,901	70,347
GeneRIFs	2,000	3,777	3,777	52,512	27,487
GO Molecular Function	3,000	2,969	2,944	81,895	23,356
MicroRNA	3,000	6,590	6,590	176,766	46,161
Mouse Phenotype	3,300	7,795	7,553	290,381	52,637
Metabolites	3,500	3,692	3,577	205,468	28,617
Structural Domains	3,500	7,115	6,746	247,885	46,463
GO Biological Process	2,300	4,305	4,287	65,669	29,988

Declustering constants and node and edge counts before and after the declustering algorithm was applied on nine FANs.

The Jaccard index is biased with respect to our desired measure of similarity when comparing two lists with a large difference in size. For example, if one gene appears in 50 sets, A, and the other in 5 sets, B, but all of these 5 sets are contained within the 50 containing the first gene (B ⊂ A), the Jaccard index is 0.1, a low similarity index even though there is 100% overlap between the two genes. To correct for this we also applied the Binomial Proportions test to measure similarity between gene pairs based on their membership in gene sets. This method was applied to GMT files with a large number of genes per set. We used the z-score from a Normal approximation to the Binomial Proportion test to quantify the similarity between pairs of genes. Z-scores were calculated using the following equation:

(2) $$z=\frac{\left(\frac{a}{c}-\frac{b}{d}\right)}{\sqrt{\frac{\frac{b}{d}\cdot \left(1-\frac{b}{d}\right)}{d}}}$$

where *a* is the number of gene sets the two genes are members of, *b* is the number of gene sets gene1 is a part of, *c* is the number of gene sets gene2 is a part of, and *d* is the total number of gene sets in the GMT file. A threshold for z-scores was chosen individually for each FAN to balance gene coverage and network sparseness (Table 2). Six functional association networks were created using this method: GeneRIF, CMAP co-expression [21], transcription factor co-regulation using ChEA [22] or TRANSFAC [23], GO molecular function [24], and GeneSigDB [25]. More details about each FAN are described below.

### **Declustering algorithm**

Initially, many of the networks generated using the Jaccard index or the Binomial Proportion test were very dense, containing many interactions between highly connected genes. This made it difficult to generate specific subnetworks for input gene lists. To reduce the edge clutter of the FANs while preserving the majority of nodes and the most relevant interactions, we computed a score for each gene pair as follows:

(3) $$w=\sqrt{a}+\sqrt{b}$$

where *w* is the weight of the edge; *a* is the connectivity degree of gene1; and *b* is the degree of gene2. Scores were sorted and the highest scoring edges were iteratively removed until there was a minimal loss of nodes and maximal loss of edges (Table 2).

### **Data extraction and FAN assembly**

The Genes2FANs database contains 14 different FANs. Some FANs are made purely from human data whereas others are from data collected in mouse. All interactions taken from the mouse are converted to their human orthologs using NCBI’s homologene. Data for the miRNA network was taken from the TargetScan database [26]. Mouse phenotype gene sets were obtained from the Mouse Genome Informatics’ Mammalian Phenotype (MGI-MP) Browser [27]. The ontology of the MGI-MP Browser has a tree structure with the most general phenotypes represented by the root nodes and increasingly specific terms at each additional level down the tree. Starting at the lowest, most specific phenotypes, we merged descendents with their ancestor terms up to the fourth level of the tree producing a condensed set of relations between phenotypes and genes. For the metabolites FAN we derived a GMT file from the Human Metabolome database [28]. Structural domains and their associated Entrez gene symbols were extracted from Pfam [29] and InterPro [30]. The FANs made from GO Biological Process (BP) and GO Molecular Function (MF) terms [24] were assembled using GO Slim. Both OMIM FANs were created from the Online Mendelian Inheritance in Man (OMIM) [31] morbid map. These two GMT files were originally created from OMIM for the Lists2Networks project [32], where the expanded file includes neighboring genes in the PPI. The smallest FAN, drug target, is made using annotated FDA approved drug target relationships extracted from DrugBank [33]. The CMAP co-expression FAN is made from the Connectivity Map (CMAP) which reports drug induced gene expression signatures applied to human cancer cell lines [21]. We created a GMT file containing the top 1000 genes that either increased or decreased in expression after drug perturbation from all the experiments in the CMAP database. Each gene set has an equal size of 1000 genes per experiment in CMAP, 500 up-regulated genes, and 500 down-regulated. Data for the GeneRIF FAN was downloaded from NCBI’s gene reference into function dataset which links PubMed IDs to Entrez gene symbols based on manual curation. The transcription factors ChIP-X FAN is made from the ChEA database [22] which is already stored in a GMT-like file, where the functional terms are transcription factors profiled by ChIP-seq/chip experiments and the genes for each term are putative targets for the profiled factor in each experiment. To create a GMT file from TRANSFAC we identified putative target genes for all the human transcription factor binding matrices in TRANSFAC. We scanned the promoter regions of all annotated human coding genes from the −2000 to +500 nucleotides relative to the transcription start site (TSS) using the Patch program provided by TRANSFAC, and then set arbitrary cutoffs to associate transcription factors to their putative targets. GeneSigDB contains thousands of gene lists from supporting material tables manually curated from gene expression studies, mostly cancer related [25]. A summary of all FANs is provided in Table 1 along with node and edge counts, and network creation cutoffs. A more detailed summary of the effects of declustering can be seen in Table 2 with declustering coefficients and node and edge count listings, before and after declustering, for each of the nine declustered FANs. Additionally, the effects of the declustering algorithm on the global network topology can be seen in Additional file 1: Figure S1.

**Figure 2:**
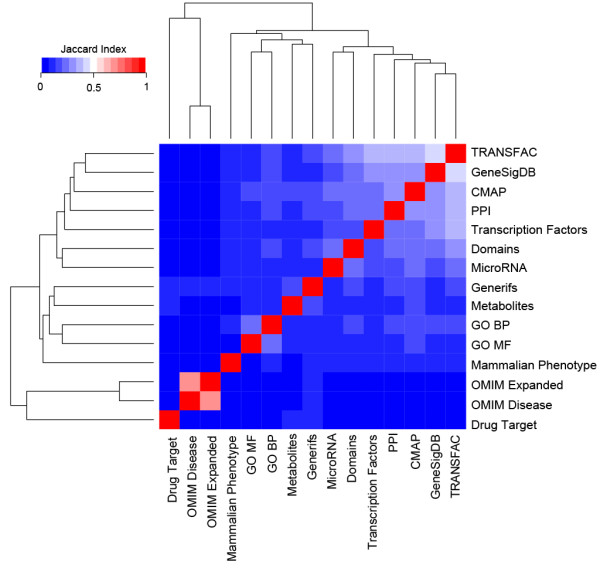
**Heatmap of genes.** Heatmap showing the similarity of the genes within each of the FANs and PPI network. Similarity was calculated using the Jaccard index.

**Figure 3:**
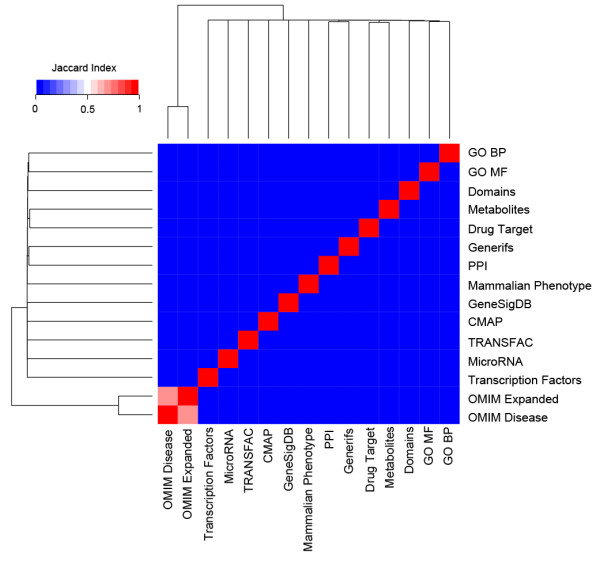
**Heatmap of edges.** Heatmap showing the similarity of the interactions connecting genes within each of the FANs and PPI network. Similarity was calculated using the Jaccard index.

**Figure 4:**
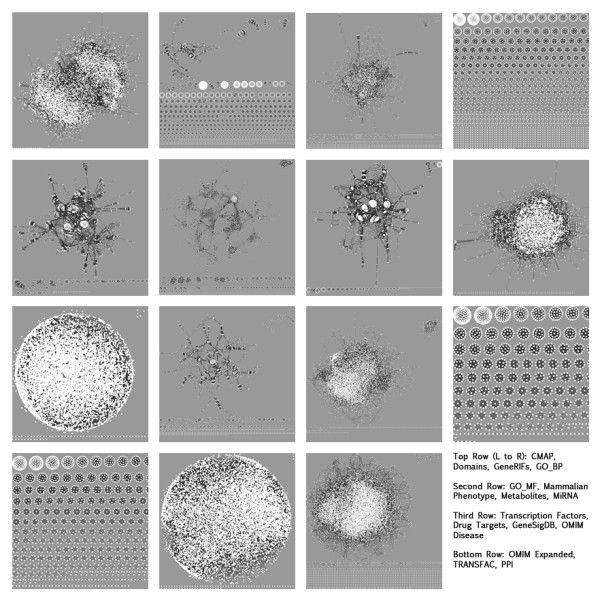
**Topology of the FANs.** The global structure of each of the FANs visualized with Cytoscape.

One of the strengths of FANs is the broad coverage of genes and their interactions. Thus, to quantify the overlap between the different types of FANs we assessed their similarity both at the gene and interaction levels, as well as comparing the FANs to the PPI network (Figures 2 and 3). Similarity was measured using the Jaccard index of the total genes and undirected edges in each of the FANs. Unsurprisingly, the largest FANs: ChEA, TRANSFAC, GeneSigDB, CMAP, PPI, and domains, contain many common genes (Figure 2). The diversity of the FANs can also be seen from the network visualization plots. Most of the networks have a large highly connected component while some networks clearly display a modular structure (Figure 4 and Additional file 1: Figure S1).

### **Developing the mammalian protein-protein interaction network**

The protein-protein interaction network used in Genes2FANs contains physical interactions between proteins reported in the literature based on experimental evidence. For Genes2FANs we consolidated 13 databases and several published studies listing experimentally verified physical protein-protein interactions. Protein-protein interactions were combined from the following sources: MINT [34], InnateDB [35], NCBI-HPRD [36], KEGG [37], IntAct [38], BioGRID [39], PPID [40], BIND [41], DIP [42], Ma’ayan et al. [43], Stelzl et al. [44], Rual et al. [45], and Yu et al. [46]. Since high-throughput studies may contain higher degree of false positives [47] we filtered the BioGRID [39] and IntAct [38] databases to include only those interactions from studies that reported 10 or less protein-protein interactions. This removes publications that report protein interactions from mass-spectrometry proteomics and yeast-2-hybrid screens. Hence, the Genes2FANs software contains two versions of PPI datasets: filtered and unfiltered.

### **Web interface**

**Figure 5:**
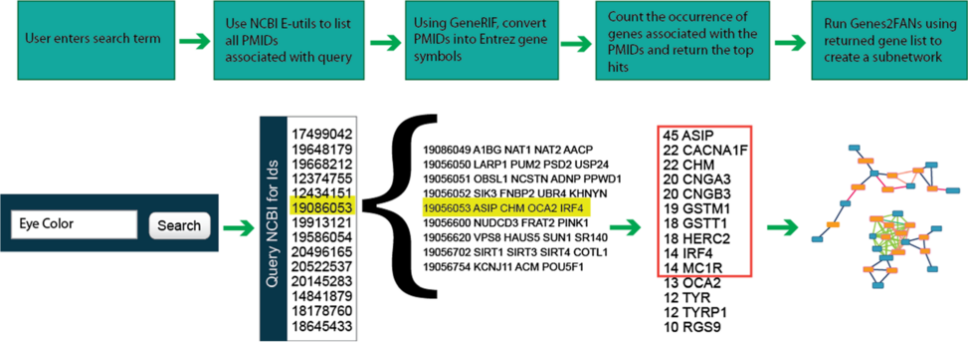
**Converting PubMed queries to lists of Entrez gene symbols.** PubMed queries are first converted into a list of PubMed IDs using NCBI’s e-utilities. For each PubMed ID a list of genes is obtained using GeneRIF. Genes are tallied and sorted by their occurrence and the top N genes are uploaded automatically into Genes2FANs.

The Genes2FANs web interface was developed using PHP, JavaScript, AJAX, and Perl. The core code for building subnetworks is implemented in C with a custom built hash function for fast access of network nodes and links. FNV, the subnetwork viewer, was implemented using Adobe Action Script 3.0 [19]. Currently, the application resides on a Linux server running Apache. To begin an analysis, users can enter a gene list by adding Entrez gene symbols one at a time or by pasting a list for upload. Results are presented to the user as an interactive subnetwork diagram and a table containing intermediate genes with z-scores indicating how significant the intermediates are for the input gene list. The interactive resultant subnetwork allows users to reposition nodes, hover over edges to reveal the gene sets that contributed to the edge, as well as pan and zoom. Users are presented with a choice of FANs to include and several options to control the size and aesthetics of the resulting subnetworks. Intermediate genes are displayed in a table ordered by their z-score computed using a Binomial Proportion test. There are also various export options allowing users to save the network for offline analysis. Figure 5 shows a screenshot of the web interface. 

### **PubMed search feature**

**Figure 6:**
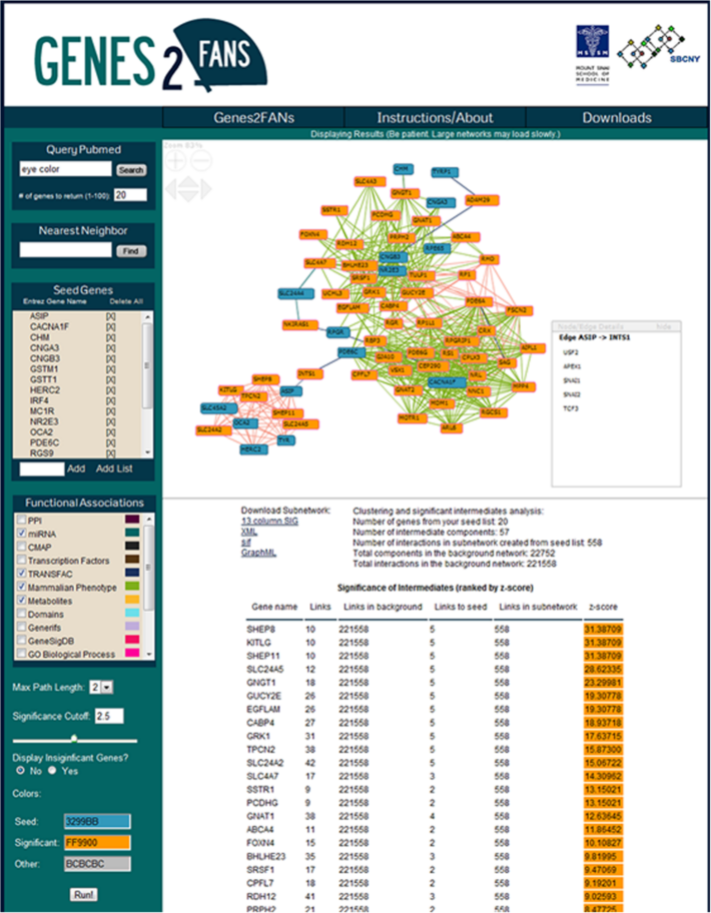
**The Genes2FANs web interface.** A screenshot showing the results of running Genes2FANs with the query “eye color”. On the left side of the page users can enter a PubMed query or a gene list and customize the output settings. The resulting subnetwork and a table listing ranked intermediates are shown on the right. Users can also obtain all the functional and binding interactions for a specific gene.

If users do not have a specific gene list to enter they can query PubMed with any search term to generate a list of genes. Genes2FANs provides users with the option to choose the number of genes to return from a PubMed search, because shorter lists are more appropriate for specific queries whereas longer lists are better for ambiguous search terms. To facilitate this function we use NCBI’s e-utilities to turn search terms into their corresponding PubMed IDs and then use the GeneRIF file to convert the PubMed IDs into human genes with occurrence counts. Genes are ranked by their number of occurrences in all returned PubMed IDs. This process is summarized in Figure 6.

## Results and discussions

### **Analysis of disease gene FAN**

To demonstrate the capabilities of Genes2FANs we applied it to find relationships between disease genes. Disease gene discovery using network approaches by pathway reconstruction has been recently proven to be very useful. Typically applications first construct a large background network and then use disease genes as seed nodes for building subnetworks that connect the seed nodes [1,48-52]. Here we implemented a similar approach to obtain a global view of subnetworks created from many disease gene lists. Using the OMIM database we compiled a list of 90 common genetic disorders. From the OMIM morbid map dataset [31] we compiled a GMT file containing all diseases with at least 10 genes (n = 90). We then used Genes2FANs to connect the genes for each disease without any intermediates using only the PPI networks or the FANs, without the OMIM FAN. We then used the disease terms from the same GMT file as input for the PubMed query tool of Genes2FANs, setting the number of returned genes to 100. The size of networks using the PPI networks only or using the FANs only (without the OMIM FAN) was then recorded. To compute the correlation between the PPI and FAN links for all the diseases, we plotted the log of the ratio of number of PPI edges against the PPI edges to functional edges. We then calculated the mean of the data points by partitioning the points into groups of 10 for the OMIM gene lists and 15 for the subnetworks made using the query PubMed function to generate a local fit. The variation was illustrated in the plot by shading the region within one standard deviation of the mean of each bin.

**Figure 7:**
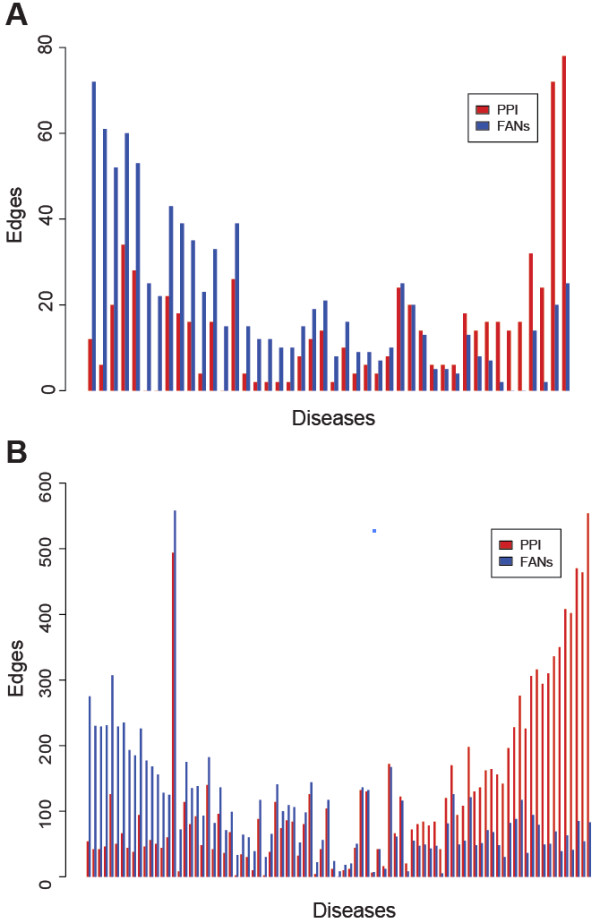
**Distribution of edges for the disease gene lists.** The distribution of edges for disease subnetworks created using genes directly from OMIM (**A**) and the disease terms with a maximum of 100 returned genes from the PubMed query tool of Genes2FANs (**B**). Diseases with a sum of PPI and functional edges less than 10 were omitted from both distribution plots.

**Figure 8:**
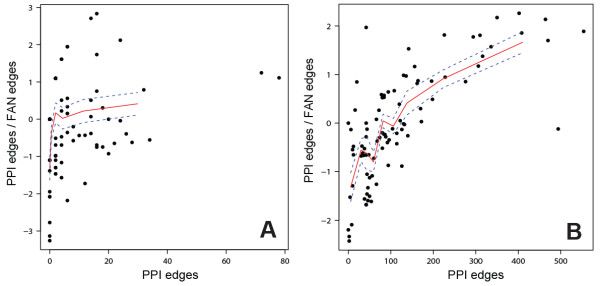
**Correlation between subnetwork size and the edge ratio of PPIs to FANs.** Scatterplots showing the correlation between the number of edges in the PPI subnetworks for each disease and the log of the ratio of PPI edges to functional edges. The red line depicts the mean of the data points (calculated by partitioning the points into groups of 10 for the OMIM disease gene lists (**A**) and 15 for the subnetworks made using the query PubMed function (**B**)). The blue dotted lines show one standard deviation away from the mean.

**Figure 9:**
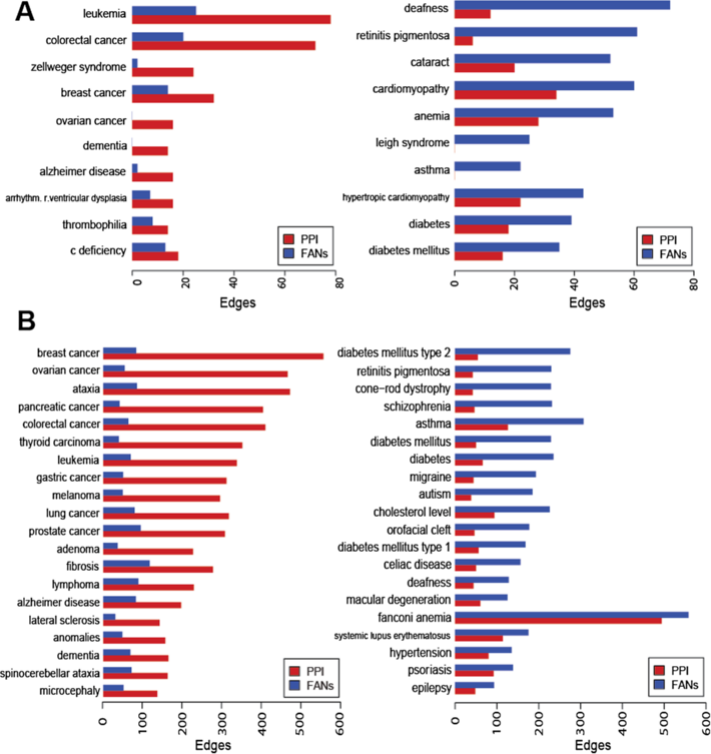
**Top diseases.** The top 10 diseases with the greatest difference in edge counts for the PPI vs. FANs disease subnetworks made from the OMIM disease gene lists (**A**) and the top 20 diseases for the subnetworks made using the query PubMed function (**B**).

With both methods, directly from OMIM or through PubMed queries, diseases show an inverse correlation between protein-protein interaction (PPI) links and other types of functional annotation links, segregating diseases with many known genes into two broad categories: those with gene products that physically interact, and those that interact functionally but not physically (Figures 7 and 8). This trend is statistically significant based on a Spearman rank correlation of 0.73 which has a p-value of 2.97×10^-10^ for the PubMed queried lists, and 0.27 for the lists directly from OMIM (p = 0.0065). The diseases that show high level of PPI and low level of functional associations include breast, ovarian, pancreatic, colorectal, thyroid, gastric, lung, and prostate cancers, as well as ataxia and leukemia (Figure 9); whereas diseases that display high level of functional interactions and low level of PPI are: deafness, type-2 diabetes mellitus, asthma, schizophrenia, autism and epilepsy. To ensure that this is not an artifact of the declustering algorithm on the FANs we ran the same process using the nine FANs before declustering. The declustering process had little effect on these results (Additional file 2: Figure S2 and Additional file 3: Figure S3) with Spearman rank correlation of 0.38 which has a p-value of 0.00026 for the PubMed queried lists, and 0.57 for the lists directly from OMIM (p = 1.99×10^-7^). The finding that some diseases have disease genes that are linked mostly through PPI, while other disease genes are mostly connected through FANs, is important because many investigations attempt to use protein interactions for novel disease gene discovery, for example, prioritizing mutations in genes detected by exome sequencing. This suggests that disease gene discovery using a PPI approach would work well for diseases such as cancers where many PPIs connect the disease gene products; however, for other complex diseases such as autism and type-2 diabetes, FANs would potentially be better for disease gene discovery.

### **Comparison to other similar tools**

Finally, we compared Genes2FANs to other similar presently available online software tools. To compute the average number of genes returned for each of the tools we used a list of 20 randomly selected human genes and calculated the average and standard deviation of unique interactions reported by each tool. We used the nearest neighbor function of Genes2FANs and summed the number of interactions returned from each of the functional networks and the PPI. For PIPs [8], we ran the tool using the default settings and counted every interaction that had a score higher than 0. We ran HEFalMP [9] to explore a gene in relation to all genes in the context of all biological processes, only counting potentially interacting genes that had a confidence score higher than 0.5. To count the number of interactions returned by GeneMania [7] we searched for human genes with default settings and counted each edge as a separate interaction. Similarly, for STRING 9.0 [5] we ran the gene query as a human gene with default settings and counted unique edges. We also tested FunCoup [10] with its default settings. By default FunCoup applies an algorithm to reduce the number of probable links for a gene query. As a result many of our queries were capped at 60 returned genes when more significant interactions were identified.

It is difficult to quantify the accuracy of our approach compared with other similar tools since there is a lack of gold standard for functional relationships between genes. As a result, we cannot fairly compare the sensitivity and specificity of our tool against existing similar tools that integrate functional relationships. To show the differences between each tool we have chosen to focus on the number of interactions that are returned for an input gene (Table 3). The totals elucidate a clear pattern; each tool is suited for different purposes. In terms of accuracy, using a tool such as GeneMania, PIPs, or FunCoup might provide a user more reliable novel PPIs likely to interact with their query. On the other hand, for a more comprehensive analysis, STRING or HEFalMP would be the best performer. It is also worth noting that there is a great deal of overlap between Genes2FANs and these existing systems. As an example, using BRCA1 as input for each of the tools with default settings, and as input for the nearest neighbor function in Genes2FANs, we observed that most of the genes returned by STRING 9.0 (10 out of 10 genes), GeneMania (12 out of 19), and FunCoup (12 out of 25) were in our PPI dataset. All but four of our functional interactions for BRCA1 were returned by HEFalMP with varying degrees of confidence and three of these functional interactions were also returned by PIPs. Those genes identified by Genes2FANs but not in HEFalMP, are OVCAS1, FAM82A1, AIMP2, and MIR21. These genes were implicated in the literature to be associated with breast and/or ovarian cancer and may be indirectly related to BRCA1.

**Table 3:** Comparison with Similar Tools

**Tool Name**	**Average Interactions**	**Background Knowledge**	**Unique Genes in database**	**Organisms**
Genes2FANs	72.1 ± 51	PPI, literature co-occurrence, miRNAs, co-regulation, domains, drug signatures & targets, gene signatures, metabolites, and phenotypes	35,078	*H.sapiens, M.musculus, R.norvegicus*
PIPs	10.1 ± 25.2	Co-expression, orthology, domains, co-localization, and PTMs	5,338	*H.sapiens*
HEFalMp	681.3 ± 1123.2	Functionally mapped data from microarray experiments and sequence comparisons	24,433	*H.sapiens*
GeneMania	78.7 ± 39.2	Co-expression, physical & genetic interactions, domains, co-localization, pathways, and orthology	155,238	*H.sapiens, A.thaliana, C.elegans, D.melanogaster, M.musculus, R.norvegicus,* and *S.cerevisiae*
STRING 9.0	24.3 ± 14.4	Co-localization, fusion, co-occurrence, co-expression, literature co-occurrence, and orthology	5,214,234	1,133 Organisms
FunCoup	47.7 ± 21.9	PPI, orthology, co-expression, miRNA, co-localization, phylogenetics, co-regulation, genetic interactions, and domains	1,800,000	*H.sapiens, M.musculus, R.norvegicus, D.melanogaster, A.thaliana, C.elegans, S.cerevisiae,*and *C.intestinalis*

Comparison of Genes2FANs with five similar tools; examining the average number of interactions returned for single gene queries, the types of background knowledge for each tool, the number of unique genes/proteins in each knowledgebase, and the supported organisms.

## Conclusions

Genes2FANs is a potentially useful tool for interpreting the relationships between gene lists in the context of their various functions and networks. Combining these functional association interactions with physical protein-protein interactions from high and low throughput datasets can be useful for revealing new biology and help form hypotheses for further experimentation. Our observation of disease gene lists commonly connected by either PPIs or FANs, but not by both, can assist with disease gene discovery strategies using network analysis and disease gene classifiers.

However, Genes2FANs is not without limitations. Currently, it does not include a confidence score for each edge. We also keep the FANs separate but all FANs can potentially be integrated into one large network. In the future we plan to constantly continue to update Genes2FANs with more FANs and to add more interactive features to the website. We also plan to develop a feature that will allow users to upload their own gene-set libraries for constructing their own functional networks. Additionally, we are working on improving our network generation process to improve the quality of the FANs.

## Availability and requirements

Project name: Genes2FANs

Project home page: http://actin.pharm.mssm.edu/ genes2FANs

Operating System: Platform Independent

Programming Language: HTML, CSS, JavaScript, Perl, C, PHP, Python, Flash/Action Script

Other Requirements: Adobe Flash Player 9.0 or higher

License: GNU GPL

## Competing interests

Authors declare that they have no competing interests.

## Authors' contributions

RD assembled the FANs and designed and implemented the web interface and database. AM designed and supervised the project and implemented the declustering algorithm. NRC helped with computing the inverse correlation and provided helpful suggestions. AM and RD wrote the paper. All authors read and approved the final manuscript.

## Supplementary Material

Additional file 1 **Figure S1.**Effect of declustering on topology. The global network structure of each of the nine declustered FANs before (left) and after (right) applying the declustering algorithm; visualized with Cytoscape. Click here for file

Additional file 2 **Figure S2.**Edge distribution of FANs before declustering. The distribution of edges for disease subnetworks created using disease genes directly from OMIM (A) and the subnetworks made using the PubMed query tool with an maximum of 100 (B) using background FANs before declustering. Diseases with a sum of PPI and FAN edges less than 10 were omitted from both bar charts. Click here for file

Additional file 3 **Figure S3.**Correlation between subnetwork size and the edge ratio of FANs to PPI before declustering. Scatterplots show the correlation between the number of edges in the PPI subnetworks for each disease and the log of the ratio of PPI edges to functional edges from subnetworks created from FANs before declustering. The red line depicts the mean of the data points (calculated by partitioning the points into groups of 10 for the OMIM disease gene lists (A) and 15 for the subnetworks made using the query PubMed function (B)). The blue dotted lines show one standard deviation away from the mean. Click here for file

Additional file 4 Table S1. Click here for file
