# Supplementary figures and images for: Genes2FANs: connecting genes through functional association networks
Source: BMC Bioinformatics. 2012 Jul 2;13:156. doi: 10.1186/1471-2105-13-156 (PMC3472228; doi:10.1186/1471-2105-13-156)

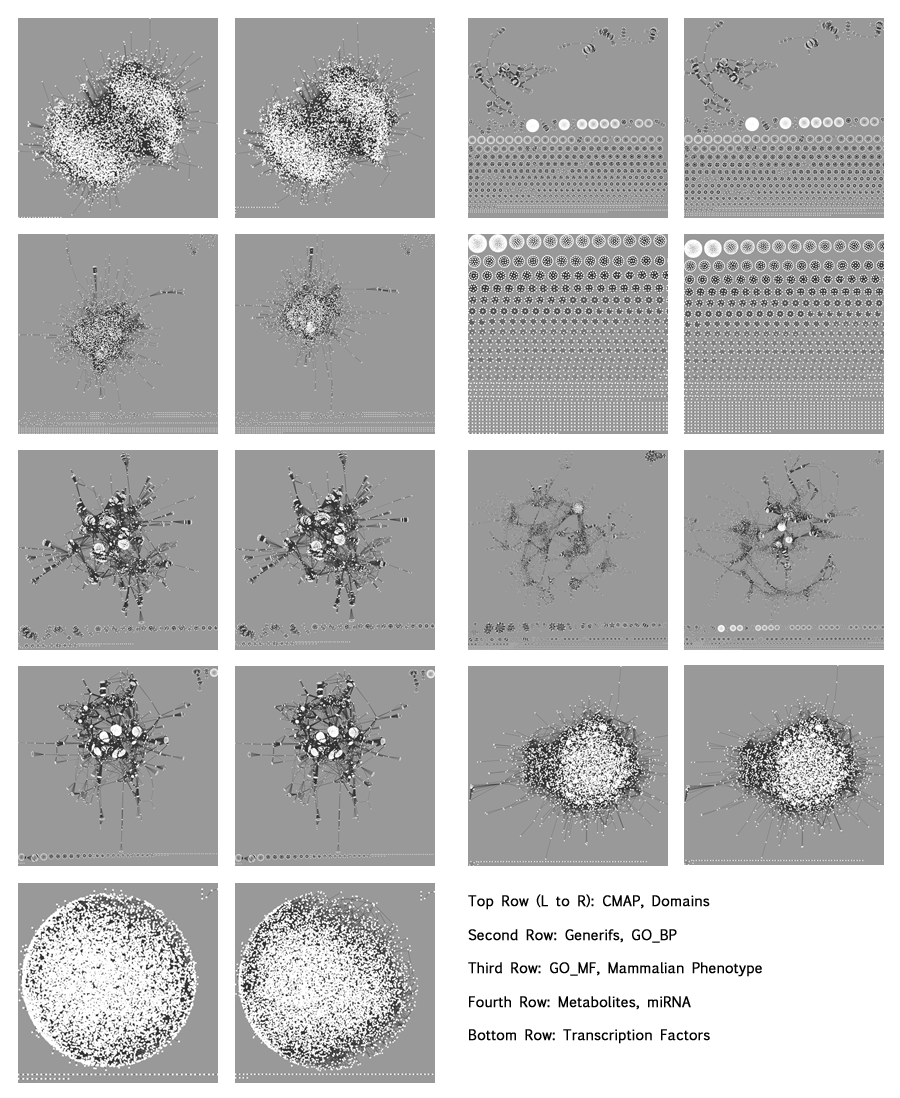

Supplement: Additional file 1 — Figure S1.Effect of declustering on topology. The global network structure of each of the nine declustered FANs before (left) and after (right) applying the declustering algorithm; visualized with Cytoscape. [file 1471-2105-13-156-S1.png]

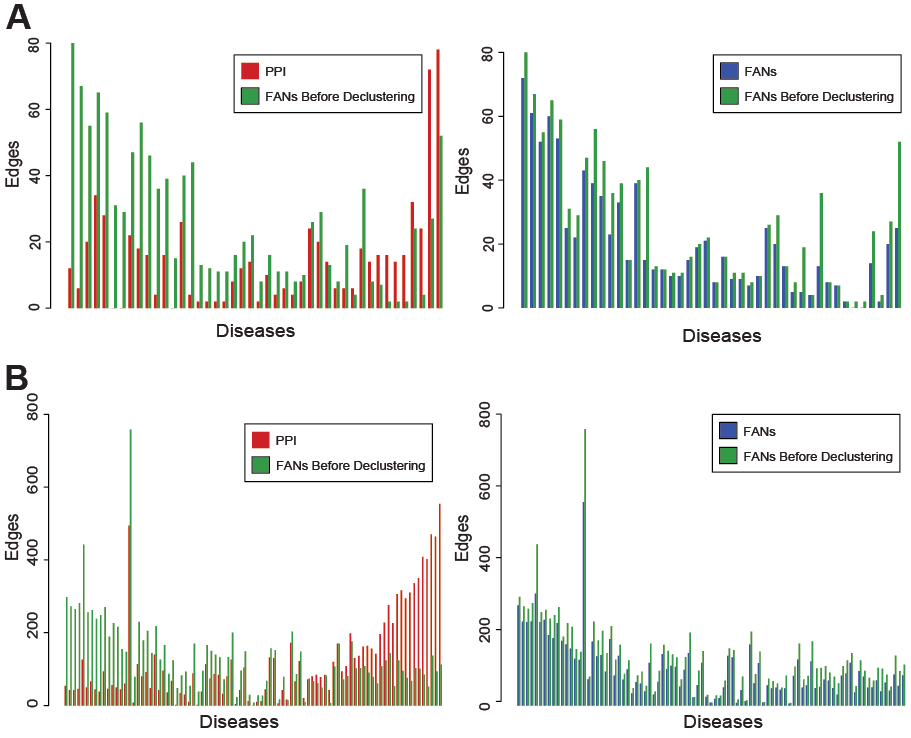

Supplement: Additional file 2 — Figure S2.Edge distribution of FANs before declustering. The distribution of edges for disease subnetworks created using disease genes directly from OMIM (A) and the subnetworks made using the PubMed query tool with an maximum of 100 (B) using background FANs before declustering. Diseases with a sum of PPI and FAN edges less than 10 were omitted from both bar charts. [file 1471-2105-13-156-S2.png]

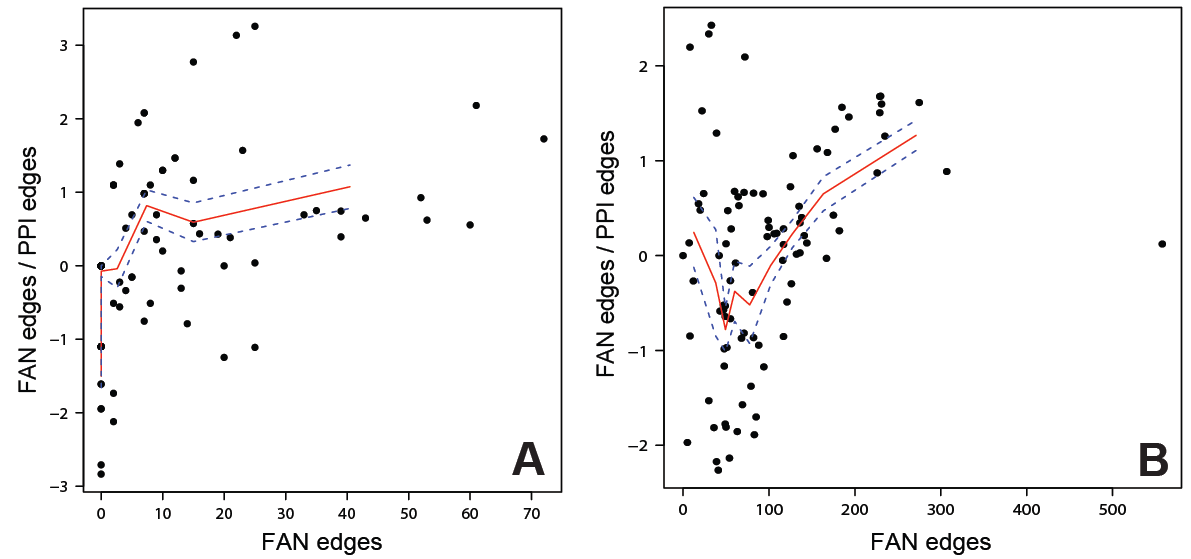

Supplement: Additional file 3 — Figure S3.Correlation between subnetwork size and the edge ratio of FANs to PPI before declustering. Scatterplots show the correlation between the number of edges in the PPI subnetworks for each disease and the log of the ratio of PPI edges to functional edges from subnetworks created from FANs before declustering. The red line depicts the mean of the data points (calculated by partitioning the points into groups of 10 for the OMIM disease gene lists (A) and 15 for the subnetworks made using the query PubMed function (B)). The blue dotted lines show one standard deviation away from the mean. [file 1471-2105-13-156-S3.png]
